# Editorial: Greening urban spaces and human health, volume III

**DOI:** 10.3389/fpubh.2026.1963120

**Published:** 2026-09-18

**Authors:** Abdullah Akpınar, Yuan Li, Ding Li

**Affiliations:** 1 Faculty of Architecture and Design, Central Asian University, Tashkent, Uzbekistan; 2 School of Geography and Tourism, Shaanxi Normal University, Xi'an, Shaanxi, China; 3 Northwest Land and Resources Research Center, Shaanxi Normal University, Xi'an, Shaanxi, China; 4 School of Public Administration, Southwestern University of Finance and Economics, Chengdu, China

**Keywords:** environmental justice, healthy aging, One Health, public health, urban green spaces

## Introduction

Urban green and blue spaces are increasingly recognized as components of public-health, climate-resilience, and social infrastructure rather than merely aesthetic amenities (1). Their importance is particularly evident as cities face interconnected pressures from population aging, mental-health burdens, environmental degradation, extreme weather, social inequality, and rapid land-use change (2, 3). From a One Health perspective, these challenges cannot be addressed by considering human health, ecological conditions, and urban development as separate domains.

This third volume of the Research Topic *Greening urban spaces and human health* brings together 27 contributions spanning public health, landscape architecture, urban planning, environmental psychology, spatial analysis, economics, governance, environmental policy, and technological innovation. The studies use diverse approaches, including multinational epidemiological analysis, panel models, systematic review, spatial econometrics, behavioral observation, social-media analysis, computer vision, machine learning, structural equation modeling, legal-text analysis, and configurational analysis.

Four interconnected messages emerge from the Research Topic. First, green and blue environments are heterogeneous exposures whose effects depend on environmental characteristics, context, and population. Second, increasingly sophisticated measurement can reveal mechanisms that conventional indicators overlook, although methodological innovation does not eliminate the need for stronger causal designs. Third, equity and healthy aging require attention to accessibility, quality, activity spaces, and differences among population groups. Finally, the health value of environmental improvement is inseparable from governance, economic incentives, land-use decisions, technological innovation, and ecological management.

## Green and blue environments are heterogeneous exposures

A central finding of the Research Topic is that “green space” should not be treated as a single exposure. Residential greenness, suburban green space, parks, streetscape vegetation, waterfronts, and green infrastructure represent different environmental conditions and should be evaluated accordingly.

Cao et al. examine residential greenness using NDVI and EVI measures among more than one million women of reproductive age in 40 low- and middle-income countries. Higher residential greenness was consistently associated with lower anemia risk, although subgroup differences were observed and the observational design does not establish causality. Ma et al. further demonstrate that the psychological value of urban and suburban green spaces is context-dependent: suburban green spaces were not significantly associated with reduced depression under normal conditions, but their benefits became more evident during restricted mobility and elevated social stress.

The Research Topic also extends beyond terrestrial green space. Li Z. et al. examine urban waterfronts as blue spaces and show that different water-body types provide distinct sensory, emotional, and social experiences. Their analysis of youth-generated social-media content highlights the importance of seasonality, including the restorative characteristics of frozen winter landscapes. Within parks, Liu B. et al. show that particular landscape features—including trees, pavements, shrubs, resting facilities, and ground characteristics—are associated with different intensities of exercise, leisure, and social behavior. Huang et al. similarly demonstrate that streetscape vegetation should not be equated with park greenness: street-level environmental features, including the Green View Index, openness, and enclosure, are associated with emotional responses in non-linear ways.

Together, these studies suggest that the quantity of vegetation alone is insufficient to characterize exposure. The principal limitation is the heterogeneity of exposure definitions across studies, which complicates comparison. Future research should therefore report environmental type, spatial scale, quality, accessibility, seasonality, and patterns of use rather than relying exclusively on area or proximity measures.

## From measurement innovation to health mechanisms

A second collective message concerns the value and limitations of methodological innovation. Liu B. et al. demonstrate how computer vision and behavioral observation can identify the specific locations and landscape characteristics associated with health-related activities. Huang et al. use street-view imagery, semantic segmentation, machine learning, GIS, and explainable artificial intelligence to identify environmental-emotion relationships that would be difficult to capture using conventional land-cover indicators. These approaches can make evidence more actionable for designers and park managers.

Spatial and longitudinal approaches further reveal mechanisms operating beyond individual sites. Zheng et al., using data from 300 Chinese prefecture-level cities, find that urban green space is associated with population aging through local effects and spatial spillovers, with benefits intensifying after a critical green-space coverage threshold. Song et al. report short-term improvements in distress, resilience, and family relationship quality following a forest-based rehabilitation program for households affected by occupational injury. However, the single-group pre–post design and absence of long-term follow-up mean that these changes should be interpreted as associations rather than confirmed causal effects.

The methodological diversity of the Topic also includes configurational and institutional approaches. Ran and Qijia identify three configurations associated with national green innovation capacity: government leadership combined with R&D investment, clean-energy supply and demand, and renewable-energy development combined with environmental greening. These pathways differ across China, Japan, and South Korea, illustrating that environmental improvement may require different combinations of enabling conditions.

The main limitation remains the gap between sophisticated measurement and causal evidence. Many studies are observational, while intervention studies may lack comparison groups or long-term follow-up. Future research should combine advanced exposure assessment with natural experiments, quasi-experimental designs, repeated outcome measurements, objective physiological or environmental indicators, and longer follow-up periods. Qualitative evidence should also be retained to capture safety, cultural meaning, perceived quality, and lived experience.

## Equity and healthy aging

A third collective finding is that environmental benefits are not distributed equally. Equity concerns not only the amount of green space available but also its quality, accessibility, safety, usability, cultural relevance, and relationship to people's everyday activity spaces.

Liu S. et al. address this Research Topic directly by moving beyond residential proximity and examining activity spaces. Their structural equation model shows that green-space quality influences exposure and perceived social benefits, which in turn contribute to perceived green justice and subjective wellbeing. The pathways remain significant across different activity-space groups, although economic conditions influence exposure in the mid-distance group.

Healthy aging is another major component of the Research Topic. Wang find that urban green spaces are positively associated with healthy aging and physical activity and that physical activity partially mediates this relationship. Importantly, the strength of the association differs between higher- and lower-income regions. Zhang J. et al. show that specific perceived qualities of urban green spaces—including cultural character, social opportunities, species richness, and serenity—are positively associated with mental restoration among older adults, with differences by age and gender.

The systematic review by Chen et al. reaches a complementary conclusion from the broader built-environment literature: greater green-space provision, better facility accessibility, and shorter travel distances are generally associated with better mental health among older adults. However, only four of the 21 included studies were longitudinal, highlighting the limited causal evidence. Wang et al. provide additional longitudinal evidence from smart older-adult care communities, where greater green-infrastructure coverage is associated with lower disease, depression, and anxiety and with greater wellbeing and social interaction. Air-quality improvement and temperature regulation appear to be important pathways, while accessibility and equitable distribution strengthen the benefits.

Equity should also extend beyond conventional urban parks. Meng and Yan demonstrate that comprehensive land consolidation can improve rural human-settlement environments by influencing agricultural productivity, land use, infrastructure, irrigation, and ecological restoration. Thus, healthy environmental planning should consider the wider spatial systems in which people live rather than limiting attention to municipal green-space provision.

The principal limitation across this theme is the scarcity of longitudinal, quasi-experimental, and distributional evaluations. Future research should assess both average benefits and subgroup effects and should monitor whether environmental interventions reduce or unintentionally widen inequalities.

## From environmental interventions to governance and sustainable urban systems

The final theme concerns implementation. The Research Topic shows that healthier environments depend on governance, finance, technology, industry, land use, ecological protection, and infrastructure—not only on the provision of parks.

Guo et al. demonstrate that green-city governance is influenced by legal strength, interdepartmental coordination, and public participation, with non-linear associations between green-space coverage and health outcomes. Their comparison of Beijing, Hangzhou, and Yinchuan illustrates how policy implementation can shape the environmental and health consequences of urban greening. Liu and Zhang broaden the perspective by framing green infrastructure as part of integrated blue–green–gray systems for flood management. Such systems can support stormwater retention, water quality, microclimate regulation, biodiversity, and climate resilience, but challenges remain in scaling, connectivity, and monitoring.

Several contributions examine the economic and technological systems that shape environmental sustainability. Li and Wang find that industrial upgrading in Chinese industrial parks can increase firm-level new-quality productivity through R&D investment and reduced information asymmetry, with heterogeneous effects according to firm and city characteristics. Resta et al. show that mental health remains insufficiently integrated into mainstream ESG frameworks and identify the rule of law as an important governance factor in their cross-national analysis. Wu and Ren show that intelligent technologies can improve the environmental performance of public-art installations in low-carbon cities, while emphasizing trade-offs involving technical cost, maintenance, durability, and user satisfaction.

The Topic also highlights green innovation and economic policy. Li finds that green innovation efficiency contributes to the development of a green low-carbon circular economy in Chongqing and is associated with improved environmental quality. Li W. et al. identify differentiated pathways for green low-carbon development across Chinese regions, emphasizing that strategies should correspond to regional developmental stages. Shi and Ge show that government subsidies and tax incentives promote green technological innovation and enterprise development, while regional differences affect policy effectiveness. Zhang B. et al. similarly find that green credit improves corporate green total-factor productivity through R&D investment and public supervision.

Other studies demonstrate the importance of spatial and ecological governance. Ge and Shi report that green spaces in Chongqing have declined overall, with industrial development and economic growth contributing to this reduction while government planning and public investment support protection. Cui finds that ecotourism in Shennongjia National Park stimulates economic growth but also increases ecological pressure, including vegetation degradation and water-quality decline, demonstrating the need to balance tourism capacity with ecological restoration. Liu L. et al. identify imbalances between ecosystem services and urbanization in western border counties and show that tourism, ecological areas, and government action can interact to influence coordination. Finally, Jiang finds continuing improvements in the green development of coastal manufacturing but identifies persistent regional differences and the need for stronger green systems, innovation, policy support, and resource allocation.

Collectively, these studies demonstrate that environmental health is shaped by upstream institutional and economic systems. Their principal limitation is that economic, governance, ecological, and direct health outcomes are still rarely evaluated within the same empirical framework. Future research should therefore connect policy and investment decisions with measurable environmental, behavioral, and health outcomes and should explicitly assess maintenance costs, implementation capacity, and distributional consequences.

## Conclusion

The 27 contributions in *Greening urban spaces and human health, volume III* collectively show that urban nature can support physical health, psychological restoration, physical activity, social interaction, healthy aging, ecological resilience, and sustainable development. However, the benefits are neither automatic nor uniformly distributed. Residential greenness, suburban green space, parks, streetscape vegetation, waterfronts, and green infrastructure represent different environmental exposures, and their effects depend on quality, accessibility, spatial configuration, seasonality, social context, and population characteristics.

The Research Topic also suggests that the next generation of research should move beyond the question of whether green space is beneficial. More important questions are which environmental characteristics matter, through which mechanisms, for whom, under what conditions, and at what spatial and temporal scales. Methodological innovation should therefore be accompanied by stronger causal designs and longer-term evaluation.

For cities, these priorities can be translated into a practical monitoring framework. Relevant indicators include equitable access and distribution, maintenance quality, safety, usability and accessibility, thermal comfort, biodiversity, physical activity and patterns of use, social participation, perceived social benefits, physical and mental health outcomes, and long-term distributional effects. For integrated blue–green–gray systems, cities should additionally monitor stormwater performance, water quality, ecological connectivity, and maintenance requirements.

One Health provides a useful overarching perspective because the studies collectively connect human wellbeing with ecological functions, biodiversity, water systems, climate adaptation, land use, and governance. The ultimate challenge is therefore not simply to increase the amount of urban nature, but to create environmental systems that are accessible, functional, ecologically sustainable, socially equitable, and resilient over time. Achieving this goal will require collaboration among public-health professionals, planners, landscape architects, environmental agencies, economists, legal scholars, technology specialists, and communities.
